# (Re)assessment of the COSMED Quark CPET and VO2Master Pro Systems for Measuring Pulmonary Gas Exchange and Ventilation

**DOI:** 10.1111/sms.70019

**Published:** 2025-01-29

**Authors:** Johan S. Thiessen, Nasimi A. Guluzade, Robin Faricier, Daniel A. Keir

**Affiliations:** ^1^ School of Kinesiology The University of Western Ontario London Ontario Canada; ^2^ Lawson Health Research Institute London Ontario Canada; ^3^ Toronto General Research Institute Toronto General Hospital Toronto Ontario Canada

**Keywords:** breathing frequency, carbon dioxide production, indirect calorimetry, metabolic cart, minute ventilation, oxygen uptake, tidal volume

## Abstract

We assessed the validity, reliability, and transferability of gas exchange and ventilatory variables from two commonly used metabolic measurement systems (COSMED Quark and VO2Master Pro). Two identical devices from each system were independently connected to a metabolic simulator (VacuMed), and 2 min of steady‐state data was recorded at simulated oxygen uptake (V̇O_2_) of 1, 2, 3, and 4 L∙min^−1^ achieved through minute ventilation (*V̇*_E_) of 30, 60, 105, and 150 L∙min^−1^. Each metabolic analyzer recorded data three times for each “intensity” in a randomized order, and assessments were completed on two separate days. Douglas bag‐based measurements were also made once at each simulated “intensity”. Measured steady‐state data (average of final 1.5 min) for both V̇O_2_ (STPD) and *V̇*_E_ (ATPS) were compared with simulated values to assess validity, repeated values between and within days assessed reliability, and between‐device comparisons provided transferability. Including both COSMED devices at all intensities, the mean percent error for V̇O_2_ was 3.5% (range: −2.5%–8.1%) and, for *V̇*_E_, was 2.0% (−0.5%–7.6%). For the VO2Master, these values averaged 0.6% (−9.3%–4.8%) and 1.1% (−6.3%–4.0%) for V̇O_2_ and *V̇*_E_, respectively. Mean percent error for Douglas Bag was 1.5%, −3.7%, −3.1%, and −2.0% for 1, 2, 3, and 4 L∙min^−1^, respectively. Between‐day differences (reliability) for V̇O_2_ of both COSMED devices ranged from −4.1% to 2.2% (mean 0.1%) and, for both VO2Masters, between −1.6% and 11.1% (mean 1.2%). Between‐device differences (transferability) ranged from −3.5% to 0.5% (mean 1.3%) for COSMED and from −11.0% to 3.6% (mean 0.0%) for VO2Master. Mean values and ranges for *V̇*_E_ were similar. When used appropriately in laboratory settings, the COSMED Quark and VO2Master Pro systems provide gas exchange and ventilatory data within an acceptable range for metabolic testing equipment that are both reliable and transferable between optimally performing devices.

## 1 Introduction

Metabolic measurement systems are frequently used in research and clinical laboratories to acquire gas exchange and ventilatory data for the assessment of aerobic fitness, metabolic function, the pathophysiology of chronic disease, and to gain further insights into muscle metabolic and cardiorespiratory responses at rest and during exercise. Metabolic analyzers measure respired flow, volume, and fractional concentrations of oxygen (O_2_) and carbon dioxide (CO_2_), from which variables such as minute ventilation (*V̇*_E_), breathing frequency (*B*_F_), tidal volume (*V*_T_), and rates of O_2_ uptake (V̇O_2_) and CO_2_ production (V̇CO_2_) are derived.

Numerous manufacturers across the globe produce metabolic measurement systems, and these devices are used in most hospitals, research, and educational exercise science laboratory settings. To highlight the increased popularity of these systems in research alone, the term “Cardiopulmonary Exercise Testing” on PubMed returns within the last decade (2014–2023), an average of 6718 research articles per year, nearly doubling that of the previous decade (2004–2013), where the annual average was 3575 publications.

For all devices, manufacturers recommend that calibration of the flow, volume, and gas sensing mechanisms is performed prior to each use. With this process, if the device software indicates approval of the calibration sequence, it is assumed by many that the measured variables of human participants will accurately reflect their “true” gas exchange and ventilation. Using a metabolic simulation device, several publications [1, 2], including a recent article by Van Hooren et al. [3], challenged this assumption. The authors simulated various *V̇*_E_, V̇O_2_, and V̇CO_2_ outputs and compared known values to those measured in 15 popular metabolic measurement systems. The results were variable, with some devices exhibiting low error (~3%–5%), whereas others showed remarkably high error (~15%–20%). Two devices on either end of this spectrum were the cart‐based COSMED Quark CPET and the portable, in‐mouth VO2Master Pro, which exhibited small and large deviations from the “true” simulated values, respectively. However, there were several notable limitations with this study. First, only one device was tested for most systems. Data from a singular device may not be “transferable” to the entire population of devices in use [4]. Second, test–retest and day‐to‐day reliability were not evaluated for all measurement systems, including the COSMED Quark. Third, information regarding how unit‐specific assumptions related to pressure, temperature, and humidity to ensure that measurement error alone was responsible for deviations from simulated values may have been incomplete.

Acknowledging these limitations, the purpose of this study was to reassess the validity, reliability, and transferability of the Quark CPET and VO2Master Pro systems by comparing their measured gas exchange and ventilatory values recorded during simulated human respiration of increasing exercise intensity. It was hypothesized that both systems would display no significant difference between measured and simulated variables acquired within‐days (validity) and that the percent error would be within both that of Douglas bag‐based measures and the acceptable range for metabolic testing equipment (i.e., ~3%, 9%, and 3% for *V̇*_E_, V̇O_2_, and V̇CO_2_, respectively [5]). We further hypothesized that the error of measurement would be repeatable between‐days (reliability, i.e., ~3%, 1%, and 1% for *V̇*_E_, V̇O_2_, and V̇CO_2_, respectively [5]) and between‐devices (transferability) at all exercise intensities. Simulated variables were compared to those measured by Douglas bag and both pairs of devices at different exercise intensities with repeated measurements within‐ and between‐days to calculate the absolute and percent error for *V̇*_E_, *B*_F_, *V*_T_, V̇O_2_, and V̇CO_2_ variables. Human experiments were also performed to compare devices at two constant intensities to determine whether between‐device differences identified from simulated experiments extend also to human data where “true” values are unknown.

## 2 Methods

### 2.1 Equipment

#### 2.1.1 Simulation of Gas Exchange and Ventilatory Variables

To produce a range of known gas exchange and ventilatory variables, a metabolic simulator with a mass flow controller was used (model 17056, VacuMed, Ventura, CA, USA). The device consists of a 3 L cylinder with a syringe connected to a crank arm and a piston disk driven by a variable‐speed motor. The piston disk can be set to manipulate the displacement of the syringe such that precise volumes of 0.5, 1.0, 1.5, 2.0, 2.5, and 3.0 L of gas are taken in (i.e., “inspired tidal volume”, *V*_TI_) and ejected per stroke (i.e., expired tidal volume, VT_E_). The motor can be set to stroke rates (i.e., “breathing frequency”, *B*_F_) ranging from 5 to 80 “breaths per minute” with an adjustable precision of 0.1 strokes per minute. Thus, a range *V̇*_E_ from 2.5 to 240 L·min^−1^ (ATPS) is achievable. The mass flow controller receives an input of gas containing 20.9% CO_2_, balance N_2_, delivered at 40–50 psi. The mass flow of this gas is manipulated by a set point module capable of delivering a flow range of 0 to 20 L·min^−1^. Mass flow is barometric pressure‐ and temperature‐adjusted to yield expired volumes of O_2_ and CO_2_ that will create precise V̇O_2_ and V̇CO_2_ (in L·min^−1^) after correction for STPD using ambient conditions. An interfacing valve system merges the respiratory (room air) and metabolic (calibration gas) flows, and a temporary expandable gas storage balloon facilitates combination of continuous inflow of the calibration gas with the tidal pattern of respiratory flows. The mixing of these two gas sources dilutes the “inspired” O_2_ (F_I_O_2_, assumed to be 20.9%) and concentrates CO_2_ (F_I_CO_2_, assumed to be 0.03%) such that fixed fractions of physiologically relevant “expired” O_2_ (F_E_O_2_) and CO_2_ (F_E_CO_2_) are produced at a respiratory exchange ratio (RER) of 1.0 and with equal VT_I_ and VT_E_, V̇CO_2_ (and V̇O_2_) is approximately equal to the mass flow of the calibration gas (L·min^−1^) multiplied by the fractional concentration of calibration gas CO_2_ (e.g., 9.70 L·min^−1^ × 0.209 = 2.03 L·min^−1^ of V̇CO_2_) [6]. The set point module is controlled to the nearest 100th of L·min^−1^ and can achieve a range of V̇O_2_ and V̇CO_2_ between 0 to 4 L·min^−1^ with reported accuracy of ± 1%. The metabolic simulator was purchased within 8 months of the described experiments and came with a certificate reporting the accuracy of the mass flow device at each set point of −0.12%, −0.33%, −0.02%, and −0.06% (for mass flows of 4.85, 9.70, 14.55, and 19.40, respectively) which is within the ±0.5% accuracy range reported for the mass flow controller.

#### 2.1.2 Equipment

Two metabolic measurement systems and the Douglas bag method were assessed. The stationary, cart‐based COSMED Quark (COSMED, Rome, Italy) was operated in breath‐by‐breath mode and consisted of a bidirectional turbine (resistance: < 0.6 cmH_2_O/L/s at 14 L/s) and an optical reader for the assessment of inspired and expired gas volumes and sampling line attached to a dual gas analyzer for the assessment of fractional concentrations of respired O_2_ (paramagnetic sensor) and CO_2_ (nondispersive infrared sensor). The turbine with a sample line assembly was attached to the outlet of the metabolic simulator. Prior to each test, gas and flow calibrations were performed using a compressed gas cylinder of known concentration (16.0% O_2_ and 5.02% CO_2_) and room air and a 3 L volume syringe according to manufacturer's recommendations. Data were continuously acquired via COSMED's OMNIA software where V̇O_2_ and V̇CO_2_ are reported in STPD and ventilatory variables (*V*_T_, and *V̇*_E_) in BTPS, assuming relative humidity and temperature at the mouth of 100% and 34°C, respectively.

The in‐mouth, portable VO2Master Pro (VO2 Master, Vernon, Canada) device also operated in breath‐by‐breath mode and consisted of a differential pressure flow sensor and O_2_ analyzer (galvanic fuel cell sensor). Three flow meters designed to measure variable *V̇*_E_ (large: 40 to 220 L·min^−1^, medium: 30 to 160 L·min^−1^ and resting: 5 to 40 L·min^−1^) are provided, and respired O_2_ is measured directly at the mouth. A one‐point calibration using room air and 3 L syringe was used to calibrate the O_2_ and flow sensors, respectively. Gas exchange (V̇O_2_ only) and ventilatory variables were collected, via Bluetooth, using the VO2Master mobile app operating in “Metabolic Simulator mode”, which reports gas exchange variables in STPD (using device‐measured ambient conditions) and ventilatory variables in ATPS.

For both the COSMED and VO2Master systems, two devices were assessed. All devices were purchased within the last 3 years, functioning properly, and all components (e.g., sampling lines, turbines, and flow meters) were brand new.

The Douglas bag approach was also performed. Briefly, expired gases from the metabolic simulator were collected in a 170 L nondiffusing bag (VacuMed, USA) that was attached to the simulator via a three‐way valve (Hans Rudolph, Kansas, USA) and a two‐way, non‐rebreathing valve (Hans Rudolph, Kansa, USA), assembled in series. The three‐way valve was opened to the bag during the “inspiratory phase” of the metabolic simulator to begin data collection, and after a period of ~2 min, it was closed at the end of full “expiration”. The total bag volume was determined by multiplying VT_E_ by *B*_F_ and collection time, whereas F_E_O_2_ and F_E_CO_2_ were determined by mass spectrometry (MGA 1100 Rio+, MA‐tech, St. Louis, USA).

### 2.2 Protocol

#### 2.2.1 Simulated Experiments

Each metabolic measurement system was tested over two consecutive days at simulated V̇O_2_ values of 1.00, 2.00, 3.00, and 4.00 L∙min^−1^. *V̇*_E_ was set to 30, 60, 105, and 150 L∙min^−1^ at each successive V̇O_2_, and these were achieved by VT_E_ and *B*_F_ of 2.0 L and 15 breaths∙min^−1^ (30 L∙min^−1^), 2.5 L and 24 breaths∙min^−1^ (60 L∙min^−1^), 3 L and 35 breaths∙min^−1^ (105 L∙min^−1^), and 3 L and 50 breaths∙min^−1^ (150 L∙min^−1^). Ventilatory values were selected based on group mean responses to exercise near these V̇O_2_ values from data of a previous publication [7]. Values provide approximate ventilatory responses of a healthy young individual with a maximal V̇O_2_ of 4.10 L∙min^−1^ and a gas exchange threshold at 2.18 L∙min^−1^.

On each day, the four metabolic analyzers were used three times at each V̇O_2_ value organized in three rounds. Each test involved 2‐min of simulated breathing. After each test, the metabolic device was removed, and another was attached until all four had recorded 2 min of data from the simulator. These steps were repeated two more times until each device had recorded three measurements. This process was first completed at a simulated V̇O_2_ value of 1.00 L∙min^−1^ and was then repeated at V̇O_2_ values of 2.00, 3.00, and 4.00 L∙min^−1^. At the end of each round of measurements, one Douglas bag collection was also performed at each simulated V̇O_2_ for a 2 min period from which mean F_E_O_2_ and F_E_CO_2_ were recorded. Devices were calibrated between each test, and, with the exception of Douglas bag, the order of device testing was randomized at each exercise “intensity”. For the VO2MasterPro devices, the “resting” flow meter was used for the 1.00 L∙min^−1^ condition, the “medium” flow meter was attached for the 2.00 L∙min^−1^ simulation, and “large” flow meter was used for simulated conditions corresponding to 3.00 and 4.00 L∙min^−1^.

To assess the stability of measured gas exchange and ventilatory values from each device, on a separate day, each device recorded data for 20 min with the simulator set to produce V̇O_2_ of 2.5 L∙min^−1^ at *V̇*_E_ of 75 L∙min^−1^ (*B*_F_ of 30 breaths per min and *V*_T_ of 2.5 L). This value represented the median of all simulated intensities.

#### 2.2.2 Human Experiments

After providing informed consent, five healthy individuals performed five repeats of a stepwise protocol that included 4 min of cycling on an electromagnetically braked ergometer (Velotron, Quarq, SRAM, Chicago, USA) at 20 W, followed by a step‐change to 100 W for an additional 6 min and a second step‐change to 160 W for 10 min (20 min total). These power outputs were purposefully selected to limit the probability of non‐steady‐state gas exchange and ventilatory responses, consistent with severe‐intensity domain exercise [8], and to elicit submaximal steady‐state V̇O_2_ in the range of 1.5 and 2.5 L∙min^−1^ for 100 and 160 W [7], respectively. This protocol was completed five times using each device (i.e., 2 × VO2Master, 2 × COSMED Quark, 1 × Douglas Bag), each performed on separate days and in a randomized order. For the Douglas Bag experiments, expired gas volume was measured as described above, except that volume was measured by a turbine (UVM, VacuMed, USA). All participants were tested at the same time of day and taken care to consume meals of similar nutritional value each day and 2–3 h before their exercise test. The protocol was reviewed and approved by the Institutional Research Ethics Review Board for human participants (WREM: 123924).

### 2.3 Data Analysis

#### 2.3.1 Simulated Experiments

**FIGURE 1:**
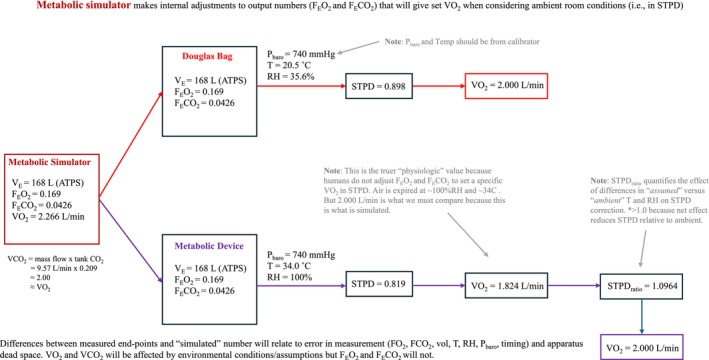
Diagram of STPD ratio, derived from metabolic simulator and metabolic analyzer STPD correction factors. The metabolic simulator makes internal adjustments to output values for expired fraction of oxygen and carbon dioxide (F_E_O_2_ and F_E_CO_2_) that will allow for a precise V̇O_2_ when considering ambient room conditions (i.e., in STPD). Therefore, if uncorrected, the differences between measured and “simulated” V̇O_2_ will relate to error in measurements for F_E_O_2_, F_E_CO_2_, ventilation (*V̇*_E_), temperature (T), relative humidity (RH), barometric pressure (Pbaro), and apparatus dead space.

The metabolic simulator makes internal adjustments to output F_E_O_2_ and F_E_CO_2_ values such that conversion from ATPS to STPD (using barometric pressure and temperature readings from its internal sensors) provides the desired V̇O_2_ and V̇CO_2_. The software of most metabolic systems apply their own STPD factors based on internal barometric pressure sensors and assumptions of expired gas temperature and humidity (assuming warm and saturated air) that are used to convert expired volumes from ATPS to STPD. However, the metabolic simulator generates volumes of expired O_2_ and CO_2_ that will achieve desired V̇O_2_ and V̇CO_2_ values in STPD assuming room temperature and humidity (i.e., cooler and dryer air). For this reason, the STPD factor applied by metabolic carts will be different than that computed by the metabolic simulator from ambient conditions. To eliminate this discrepancy, in the comparison of “simulated” versus “measured” variables, it is necessary to multiply all “measured” V̇O_2_ and V̇CO_2_ by the ratio of ambient STPD to device‐determined STPD (see Figure 1). This ratio quantifies the effect that differences in assumed versus ambient temperature/relative humidity have on STPD correction. To compare “simulated” versus “measured”, the following procedures were applied (a schematic description of steps is also provided in Figure 1):

#### 2.3.2 COSMED Quark

COSMED contains its own internal barometric pressure sensor that it uses to convert respired volumes from ATPS to BTPS for *V̇*_E_ and VT_E_ assuming a relative humidity of 100% and temperature of 34°C. Because *V̇*_E_ and VT_E_ are expressed in BTPS and this factor is provided in its breath‐by‐breath data file, we divided these values by the software‐calculated BTPS factor to retrieve ATPS values of *V̇*_E_ and VT_E_ which equate to the output of the simulator. For the calculation of V̇O_2_ and V̇CO_2_, the COSMED software converts ATPS volumes to STPD, using its measured barometric pressure and assuming a relative humidity of 100% and temperature of 34°C. However, the metabolic simulator generates volumes of expired O_2_ and CO_2_ that will achieve desired V̇O_2_ and V̇CO_2_ values in STPD assuming room temperature and humidity. Thus, the STPD factor reported by COSMED is consistently lower than that computed from ambient conditions. To control for theses differences, it was necessary to multiply the “measured” V̇O_2_ and V̇CO_2_ by the ratio of ambient STPD to device‐determined STPD (see Figure 1).

#### 2.3.3 VO2Master

In “Metabolic Simulator mode”, VO2Master Pro reports *V̇*_E_ in ATPS. Thus, no corrections were necessary to compare the measured *V̇*_E_ (and VT_E_) to the simulated values. The V̇O_2_ data are expressed in units of mL·kg^−1^·min^−1^ in STPD. Each data point was multiplied by 75 kg to obtain absolute V̇O_2_ in mL·min^−1^. VO2Master also contains its own temperature, pressure, and humidity sensors. These values are used to determine a STPD factor. The typical software output from this device (i.e., “Default, On Person mode”) does not provide the exact STPD factor (calculated from internal barometric pressure sensor and assumed humidity and temperature of 100% and 34°C, respectively). However, when operating in “Metabolic Simulator mode”, the STPD factor is computed using device‐measured humidity and temperature, and STPD‐corrected V̇O_2_ values are reported for each breath. Importantly, we observed that relative humidity, temperature, and barometric pressure registered by the metabolic simulator were not identical to those of VO2Master. To account for these differences, the ambient STPD factor (as derived by the simulator) was divided by the VO2Master STPD factor, and V̇O_2_ associated with each breath was multiplied by this ratio to determine “true” measured values.

#### 2.3.4 Douglas Bag Method

During simulations, *V̇*_E_ in ATPS is known based on the frequency of “breaths” delivered, the syringe volume, and time. *V̇*_E_ was converted to STPD using the temperature and barometric pressure readings from the simulator and humidity readings from a laboratory‐grade hygrometer (VWR Traceable Excursion‐Trac USB Datalogging Dual Hygrometer, Radnor Corporate Center, Radnor, PA, USA.). Next, V̇O_2_ was calculated using the Haldane equation:
(1)

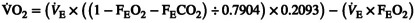

where V̇O_2_ is the rate of oxygen consumption (in L·min^−1^), *V̇*_E_ is the expired minute ventilation (L·min^−1^ STPD), F_E_O_2_ is the fractional expired concentration of oxygen (in %), and F_E_CO_2_ is the fractional concentration of carbon dioxide (in %). V̇CO_2_ was calculated as
(2)

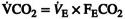




For all trials, the average of data corresponding to “breaths” appearing in the last 90 s was used to compare “measured” to “simulated” data.

For the 20 min trials, V̇O_2_ and *V̇*_E_ were computed over 90 s intervals beginning at 30 s, 600 s, and 1100 s. Data from each device were fit with a linear regression, and the corresponding slopes were compared to zero.

### 2.4 Statistical Analysis

Data are presented as mean ± SD. Error (∆) was assessed in original units (“measured” – “expected”; referred to as “error”), as a percent difference ([measured – “expected”] ÷ “expected” × 100; referred to as “percent error”) and were also expressed as absolute values (i.e., magnitude of error without directional bias referred to as “absolute error” and “absolute percent error”). To assess device validity, the original units (mL·min^−1^) and relative (%) error computed from the difference between “measured” and “simulated” data were compared. To assess between‐day reliability, coefficient of variance (CV), two‐way mixed effects intra‐class correlation coefficient (ICC), and mean bias with 95% confidence intervals were computed for each device at each “intensity”. For each device and intensity, the mean bias was calculated as the mean between‐day difference (Day 1 minus Day 2) with the mean for each day computed from the average of the three within‐day measures. Within days, a one‐way analysis of variance (ANOVA) was completed. For transferability, a two‐way (trial × intensity) ANOVA, unpaired t‐test, and mean bias with 95% confidence intervals were performed. For human trials, steady‐state gas exchange and ventilatory values obtained from each device and the Douglas bag method were compared by a one‐way repeated measures ANOVA. All statistical analyses were completed using SPSS (Version 29.0.2.0, IBM), with significance determined at *p* < 0.05.

## 3 Results

All collected data for all trials are available in an accompanying supplement (File S1).

### 3.1 Validity

At a simulated V̇O_2_ of 1000, 2000, 3000, and 4000 mL·min^−1^, Douglas bag‐derived measures of V̇O_2_ were 1015, 1926, 2908, and 3920 mL·min^−1^, respectively, or 1.5%, −3.7%, −3.1%, and −2.0%, different from expected.

**FIGURE 2:**
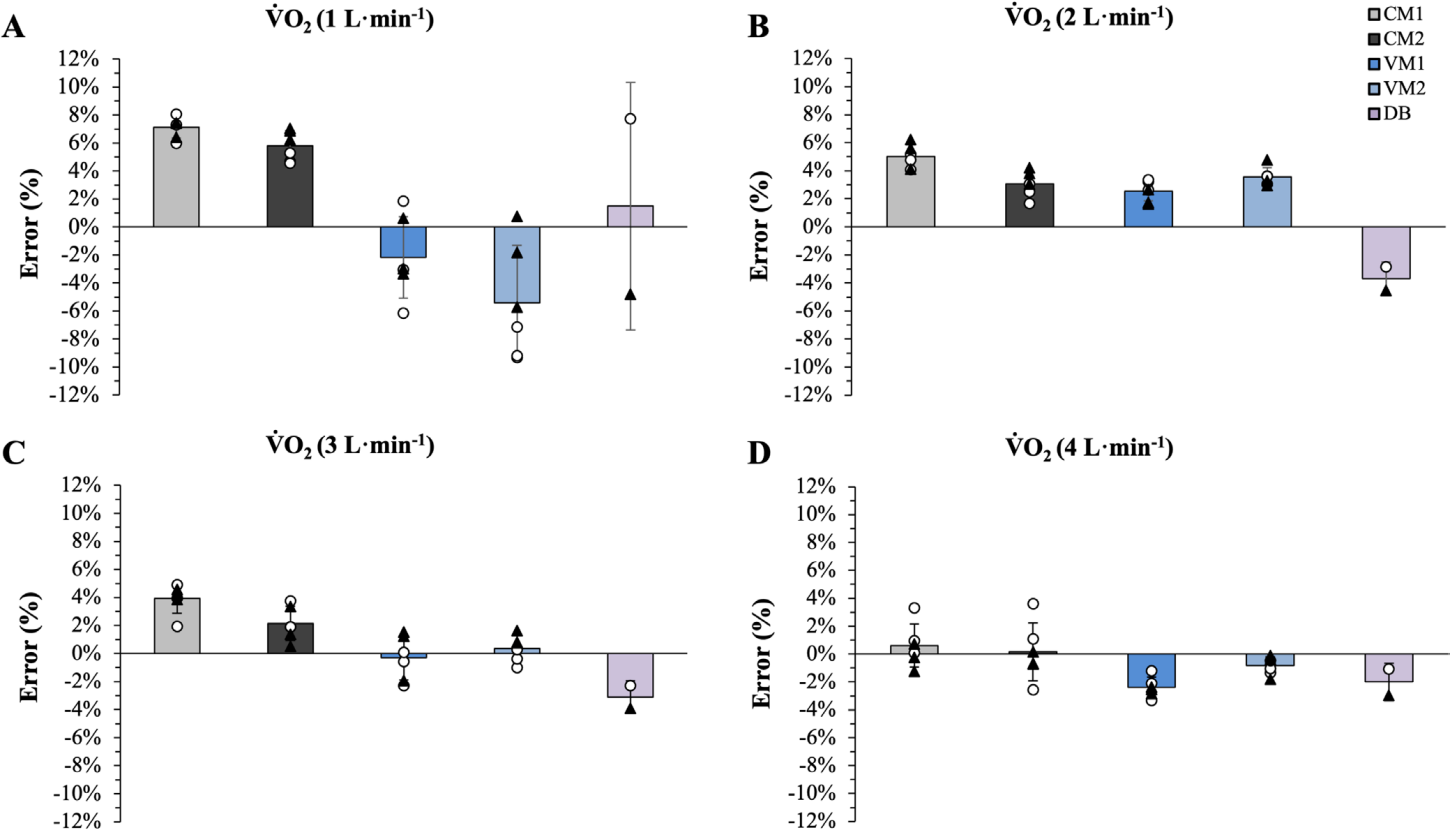
Mean error for each metabolic measurement device and the Douglas bag method is plotted for simulated V̇O_2_ intensity of 1.0 (A), 2.0 (B), 3.0 (C), and 4.0 L·min^−1^ (D) with individual trials for Day 1 and Day 2 also displayed. CM1 and CM2 refer to COSMED Quark CPET devices 1 and 2, VM 1 and VM2 refer to VO2Master Pro devices 1 and 2 and DB refers to the Douglas bag method.

**FIGURE 3:**
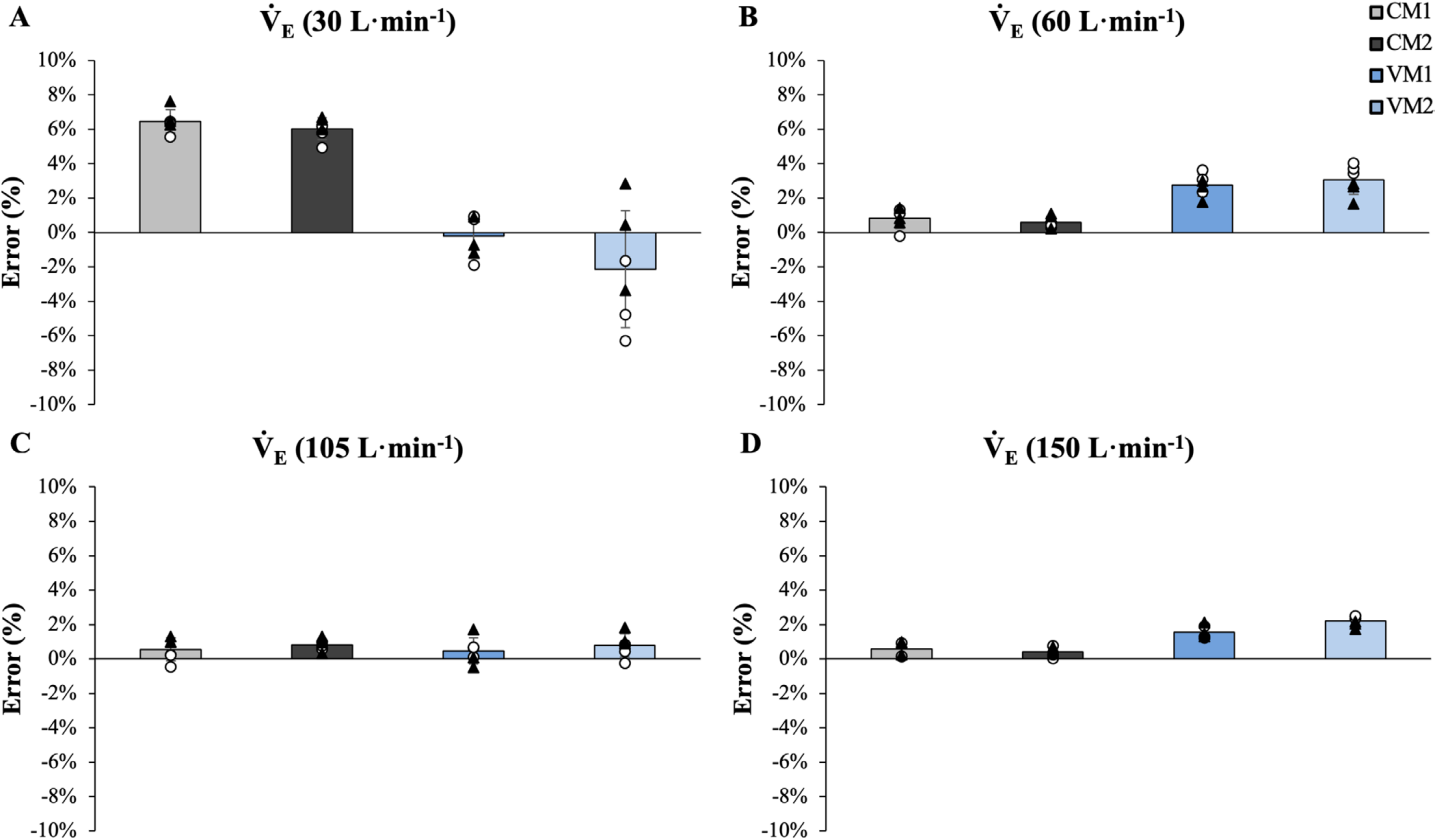
Mean error for each metabolic measurement device and the Douglas bag method is plotted for simulated *V̇*_E_ of 30 (A), 60 (B), 105 (C), and 150 L·min^−1^ (D) with individual trials for Day 1 and Day 2 also displayed. CM1 and CM2 refer to COSMED Quark CPET devices 1 and 2, VM 1 and VM2 refer to VO2Master Pro devices 1 and 2 and DB refers to the Douglas bag method.

With respect to the two metabolic measurement systems, mean absolute error was consistent among intensities for both systems, ranging from 4 to 147 mL·min^−1^ for the COSMED Quark and 4 to 132 mL·min^−1^ for the VO2Master system. The mean percent error (±SD) for the VO2Master Pro, COSMED Quark, and Douglas Bag measurements at each simulated V̇O_2_ are presented in Figure 2. For both metabolic measurement systems, excluding the Douglas bag, the mean percent error decreased as simulated “intensity” increased from 1000 to 4000 mL·min^−1^ (Figure 2). Regarding *V̇*_E_, the mean error in L·min^−1^ remained consistent across intensities for both systems. For the two COSMED Quarks, the mean percent error at 30 L·min^−1^ of *V̇*_E_ deviated from expected by 6.5% and 6.0%, equivalent to 1.94 and 1.81 L·min^−1^ but, for all other “intensities”, displayed low mean percent error values ranging from 0.42% to 0.84% or 0.62 to 0.86 L·min^−1^ (see Figure 3). For the VO2Master Pro systems, *V̇*_E_ values were consistent across intensities ranging between 0.19% and 3.07% or 0.06 and 3.33 L·min^−1^ of simulated values. The mean (±SD) error in original units and percent error of all devices and for all variables (*V̇*_E_, B_F_, *V*_*T*_, V̇O_2_, V̇CO_2_, and RER) are reported in Table 1 (Note: these values were computed for each device including both days and all trials).

**TABLE 1:** Mean error (original units) and mean absolute percent error combining all days and intensities.

Devices	∆ V̇O_2_ (mL·min^−1^)	∆ *V̇*_E_ (L·min^−1^)	∆ *B*_F_ (bpm)	∆ *V*_T_ (L)	∆ V̇CO_2_ (mL·min^−1^)	∆ RER
COSMED #1	78 ± 29	1.0 ± 0.47	0.09 ± 0.06	0.05 ± 0.01	−17 ± 20	−0.03 ± 0.01
	4.3% ± 2.5%	2.2% ± 2.6%	0.4% ± 0.2%	2.0% ± 2.6%	3.0% ± 2.4%	3.2% ± 1.5%
COSMED #2	48 ± 37	0.9 ± 0.30	0.09 ± 0.06	0.03 ± 0.01	−9 ± 41	−0.02 ± 0.01
	3.1.% ± 2.0%	2.0% ± 2.4%	0.3% ± 0.2%	1.7% ± 2.5%	3.1% ± 2.2%	1.8% ± 1.6%
VO2Master #1	−19 ± 30	1.1 ± 0.52	0.07 ± 0.06	0.03 ± 0.02	—	—
	2.3% ± 1.3%	1.5% ± 0.9%	0.3% ± 0.2%	1.4% ± 0.9%	—	—
VO2Master #2	−1 ± 27	1.3 ± 0.67	0.06 ± 0.08	0.03 ± 0.03	—	—
	2.7% ± 2.8%	2.4% ± 1.5%	0.3% ± 0.3%	2.0% ± 1.4%	—	—

*Note:* Data are presented as mean ± SD.

Abbreviations: B_F_, breathing frequency; RER, respiratory exchange ratio; V̇CO_2_, carbon dioxide production; *V̇*_E_, minute ventilation; V̇O_2_, oxygen consumption; *V*_T_, tidal volume.

### 3.2 Reliability

**FIGURE 4:**
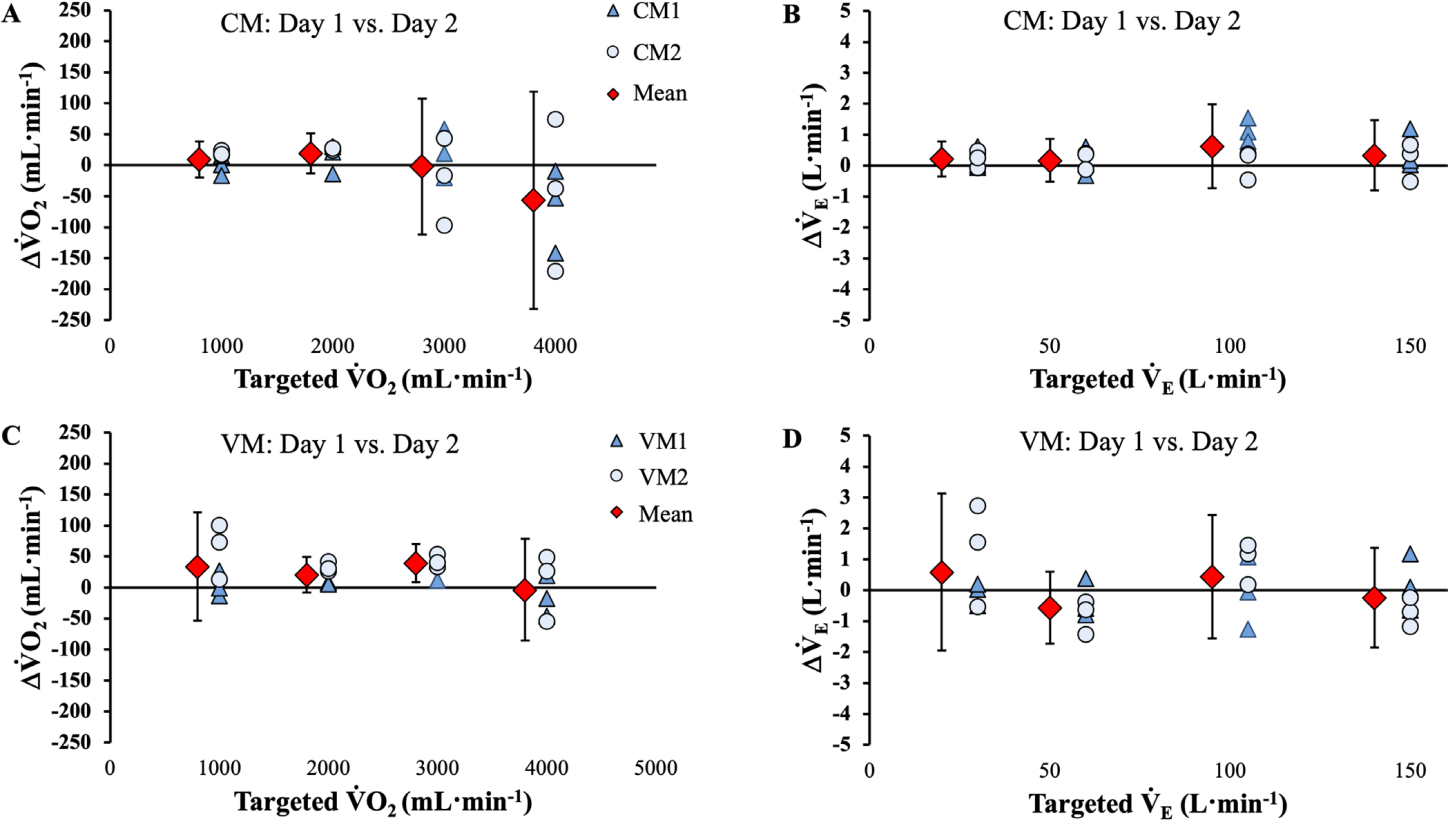
Plots displaying between‐day differences for the COSMED Quark (CM) and VO2Master Pro (VM) metabolic measurement systems at all simulated V̇O_2_ and *V̇*_E_ outputs. The mean bias including both devices (i.e., CM1 and CM2 and VM1 and VM2) are displayed (red diamond), with vertical error bars representing the 95% confidence interval specific to each simulated “intensity”. Individual data points representing between‐day and like‐trial differences are also displayed. Panels (A) and (B) display the day‐to‐day error for V̇O_2_ and *V̇*_E_, respectively, of the COSMED Quark devices. The day‐to‐day error for the VO2Master Pro devices are depicted in Panels (C) and (D) for V̇O_2_ and *V̇*_E_, respectively.

Mean bias analyses of between‐day differences for V̇O_2_ and *V̇*_E_ measured by all four devices at each simulated exercise intensity are displayed in Figure 4. Between‐days, the mean percent error for V̇O_2_ and *V̇*_E_ remained reasonably consistent across the simulated intensities for both metabolic measurement systems. For V̇O_2_ mean percent error, the COSMED Quark varied by 0.01%–1.79% between‐ and within‐days whereas, for the VO2Master Pro, variability ranged between 0.19% and 6.92%. For *V̇*_E_, the measured test–retest percent errors were 0.22%–0.68% and 0.15%–1.92% for COSMED Quark and VO2Master Pro, respectively. The mean error (in original units), 95% confidence intervals, and ICC for each device at each intensity are presented in Table 2. The Douglas bag‐derived V̇O_2_ measurements displayed a variance of 11.62%, 1.73%, 1.64%, and 1.92% at 1.0, 2.0, 3.0, and 4.0 L·min^−1^.

**TABLE 2:** Between‐day mean bias, standard deviation, and 95% confidence intervals for each device at each intensity.

Variables		1 L·min^−1^		2 L·min^−1^		3 L·min^−1^		4 L·min^−1^	
		CM	VM	CM	VM	CM	VM	CM	VM
V̇O_2_	Mean bias	9	34	19	‐8	−2	40	−57	‐4
	SD	15	44	17	24	56	16	89	42
	Upper 5% CI	39	121	52	40	107	71	119	79
	Lower 95% CI	−20	−53	−13	−56	−112	9	−232	‐86
*V̇*_E_	Mean bias	0.22	0.59	0.17	‐0.56	0.63	0.44	0.33	‐0.24
	SD	0.29	1.30	0.35	0.59	0.69	1.02	0.58	0.82
	Upper 95% CI	0.79	3.14	0.86	0.60	1.98	2.44	1.47	1.37
	Lower 95% CI	−0.35	−1.95	−0.53	−1.72	−0.73	−1.55	−0.80	−1.85

*Note:* The mean bias was computed from the average of all between‐day differences including both devices.

Abbreviations: 95% CI, 95% confidence interval; CM, COSMED Quark; *V̇*_E_, minute ventilation; VM, VO2Master Pro; V̇O_2_, oxygen consumption.

### 3.3 Transferability

**FIGURE 5:**
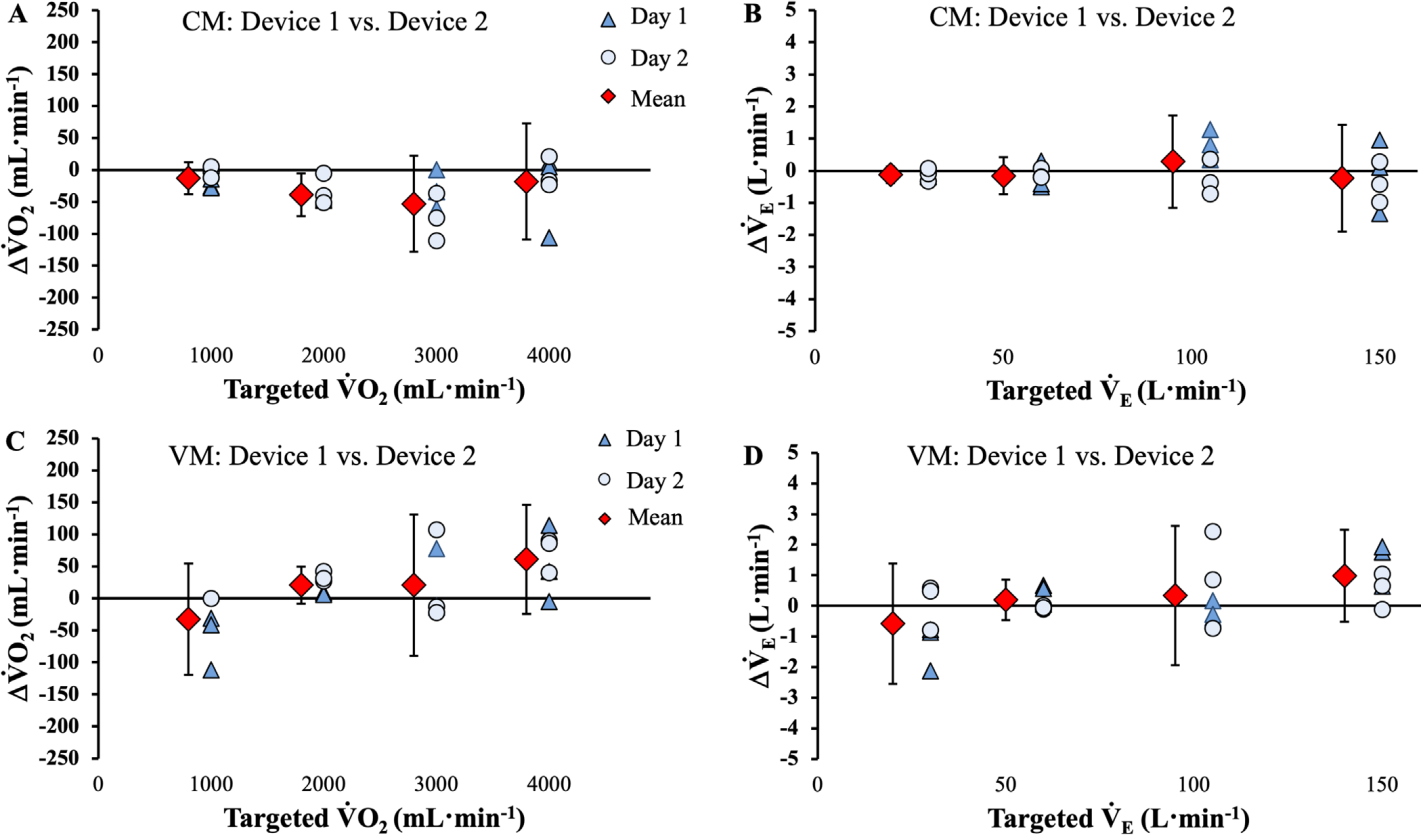
Plots displaying between‐device differences for the COSMED Quark (CM) and VO2Master Pro (VM) metabolic measurement systems at all simulated V̇O_2_ and *V̇*_E_ outputs. The mean inter‐device bias (i.e., CM1—CM2 and VM1—VM2) are displayed (red diamonds), with vertical error bars representing the 95% confidence intervals specific to each simulated “intensity”. Individual data points representing inter‐device and like‐trial differences are also displayed. Panels (A) and (B) display the inter‐device error for V̇O_2_ and *V̇*_E_, respectively of the COSMED Quark devices. The inter‐device error for of VO2Master Pro systems are depicted in Panels (C) and (D) for V̇O_2_ and *V̇*_E_, respectively.

Measured V̇O_2_ and *V̇*_E_ were compared between the two pairs of devices from the same manufacturer (Figure 5). Between‐device differences in measured V̇O_2_ for the VO2Master Pro system tended to widen linearly with increasing simulated V̇O_2_ (Figure 5C), whereas these remained relatively stable for the COSMED Quark systems (Figure 5A). For *V̇*_E_, the measured *V̇*_E_ also tended to increase linearly with the simulated conditions for the VO2Master systems (Figure 5D) but remained consistent for the COSMED Quark systems. Mean bias using original units and confidence for between‐device comparisons at each intensity are available in Table 3.

**TABLE 3:** Between‐device mean bias, standard deviation, and 95% confidence intervals for each device at each intensity.

Variables		1 L·min^−1^		2 L·min^−1^		3 L·min^−1^		4 L·min^−1^	
		CM	VM	CM	VM	CM	VM	CM	VM
V̇O_2_	Mean bias	‐13	‐32	‐39	21	−53	21	−18	61
	SD	13	44	17	15	38	57	46	43
	Upper 95% CI	12	55	‐5	50	22	131	73	146
	Lower 95% CI	−38	−119	−72	−8	−128	‐90	−109	−24
	ICC	0.99	0.99	0.99	0.99	0.99	0.99	0.99	0.99
*V̇*_E_	Mean bias	‐0.13	‐0.58	‐0.16	0.19	0.28	0.34	‐0.23	0.98
	SD	0.13	1.00	0.30	0.34	0.73	1.16	0.85	0.76
	Upper 95% CI	0.12	1.39	0.43	0.85	1.72	2.62	1.43	2.48
	Lower 95% CI	−0.38	−2.55	−0.74	‐0.47	−1.16	−1.93	−1.90	−0.52
	ICC	0.99	0.99	0.99	0.99	0.99	0.99	0.99	0.99

*Note:* The mean bias was computed from the average of all between‐device differences acquired during like‐trials.

Abbreviations: 95% CI, 95% confidence interval; CM, COSMED Quark; *V̇*_E_, minute ventilation; VM, VO2Master Pro; V̇O_2_, oxygen consumption.

### 3.4 Human Experiments

There was no between‐device difference in steady‐state V̇O_2_ measured during exercise at 100 W (*p* = 0.582) or 160 W (*p* = 0.220). Table 4 displays the mean steady‐state V̇O_2_ values measured by each system. The between‐device coefficient of variation was 6.9% at 100 W and 6.4% at 160 W. There was also no between‐device difference in steady‐state *V̇*_E_ measured during exercise at 100 W (*p* = 0.112) or 160 W (*p* = 0.069); CV was 7.6% and 8.2%, for 100 versus 160 W, respectively. Between‐participants, the mean CVs in steady‐state V̇O_2_ were 8%, 11%, and 10% for the COSMED Quark, VO2Master, and Douglas Bag measures, respectively. For steady‐state *V̇*_E_, these values were 12%, 12%, and 15%.

**TABLE 4:** Summary diagram of collected human V̇O_2_ data (*n* = 5) at 100 W and 160 W from all metabolic measurement systems and the Douglas bag method.

Intensity (W)	COSMED #1 (L·min^−1^)	COSMED #2 (L·min^−1^)	VO2Master #1 (L·min^−1^)	VO2Master #2 (L·min^−1^)	Douglas bag (L·min^−1^)	CV (%)	*p*
100	1.83 ± 0.13	1.87 ± 0.17	1.78 ± 0.14	1.73 ± 0.24	1.78 ± 0.19	6.93 ± 3.91	0.582
160	2.58 ± 0.20	2.54 ± 0.23	2.63 ± 0.30	2.69 ± 0.28	2.79 ± 0.26	6.44 ± 3.8	0.220

*Note:* Data are presented as mean ± SD. A one‐way, repeated measure ANOVA was used to generate *p*‐values.

Abbreviations: CV, coefficient of variation; V̇O_2_, oxygen consumption.

### 3.5 20‐Min Trials

Over a single, 20‐min simulated trial at 2.5 L·min^−1^, the registered V̇O_2_ from both COSMED devices did not change appreciably from the first two to final minutes (a change of 1 and − 1 mL·min^−1^·min^−1^ for devices 1 and 2, respectively). The VO2Master Pro devices exhibited positive drifts that were exponential in nature. Device 1 increased from 2.60 L·min^−1^ at the second minute to 2.68 L·min^−1^ at the 10th minute (by 80 mL·min^−1^) and to 2.71 L·min^−1^ at the 20th minute (by 30 mL·min^−1^). Device 2 increased by 140 L·min^−1^ and 30 L·min^−1^ over these same epochs from an initial value of 2.59 L·min^−1^. Measured *V̇*_E_ from both COSMED devices showed a modest increase from the first two to final minutes (a change of 0.01 and 0.02 L·min^−1^·min^−1^ for devices 1 and 2, respectively), whereas the VO2Master Pro devices exhibited a minor linear drift of 0.04 and 0.05 L·min^−1^·min^−1^ of *V̇*_E_ for devices 1 and 2 equivalent to less than 1 L·min^−1^ of *V̇*_E_.

To confirm the exponentiality of the VO2Master Pro devices, three additional 20‐min trials at simulated V̇O_2_ of 2.5 L·min^−1^ were performed. Identical behavior was noted for all six trials with plateaus occurring at 2.57, 2.56, and 2.56 L·min^−1^ for device 1 and 2.42, 2.52, and 2.56 L·min^−1^ for device 2. In five trials, the change in V̇O_2_ from the 2nd to 20th minute was between 110 and 165 mL·min^−1^ but was 0 mL·min^−1^ in one trial.

## 4 Discussion

Using a metabolic simulator, we assessed the validity, reliability, and transferability of two popular, commercially available metabolic measurement systems in a laboratory setting (COSMED Quark and VO2Master Pro). Comparisons with Douglas bag‐based measures and in human experiments were also performed. Both systems displayed a percent error of V̇O_2_ and *V̇*_E_ during simulated breathing generally below 3%, and this was consistent between‐days, between‐devices, and with Douglas Bag measures within a simulated V̇O_2_ range of 1–4 L·min^−1^. In addition, steady‐state V̇O_2_ and *V̇*_E_ measured in humans at two different cycling intensities also exhibited consistent values. These results demonstrate that in well‐controlled laboratory settings, the COSMED Quark and VO2Master Pro systems provide consistently accurate V̇O_2_ and V̇_E_ measurements that are comparable to the Douglas Bag.

### 4.1 Validity

The Douglas bag method is often considered the gold standard for gas measurement of gas exchange data in resting and exercising humans. At simulated V̇O_2_ of 1000, 2000, 3000, and 4000 mL·min^−1^, Douglas bag‐derived measures of V̇O_2_ averaged 1015, 1926, 2908, and 3920 mL·min^−1^ equivalent to percent errors of 1.5%, −3.7%, −3.1%, and −2.0%, respectively. Considering a manufacturer reported simulator accuracy of ±1% and the propensity for Douglas Bag measures to slightly underestimate V̇O_2_ due to valve assembly dead space, gas diffusion, and leaks [9], these values are nearly perfect to those simulated. Comparatively, both COSMED Quark and VO2Master Pro systems tended to overestimate V̇O_2_ with a similar absolute bias as intensity increased (see Table 1). Because the error in mL·min^−1^ remained relatively consistent as intensity increased, the percent error was progressively reduced (see Figure 2).

Like V̇O_2_, observations for *V̇*_E_ were similar. On average, differences between VO2Master Pro‐measured values and known values were −0.35 ± 0.69, 1.75 ± 0.45, 0.66 ± 075, 2.84 ± 0.49 L∙min^−1^ at 30, 60, 105, and 150 L∙min^−1^, respectively. These differences were 1.87 ± 0.20, 0.43 ± 0.29, 0.72 ± 0.53, and 0.74 ± 0.53 L∙min^−1^ for the COSMED Quark. Notably, compared to higher simulated intensities, the percent error for COSMED was greater at the lowest simulated intensity (6.5% and 6.0% for device 1 and 2, respectively) and compared to the VO2Master (see Figure 3). These overestimations in *V̇*_E_ may stem from the use of a turbine (instead of a flow meter) to measure *V*_*T*_. The lower *B*_F_ that are common at rest or low exercise intensities allow the impeller to continue to spin, even after expiration has ended.

Unlike Van Hooren et al. [3], who reported consistent underestimations of the VO2Master Pro that increased with higher simulated V̇O_2_ (by ~7% to 20% or from −73 to −794 mL∙min^−1^), the devices used in our study were much more accurate (mean percent error of ~0.6%). A key difference between studies were the approach to standardizing data for appropriate comparisons. The VO2Master converts volumes from ATPS to STPD using an internal barometric pressure sensor and assuming temperature and relative humidity at the mouth of 34°C and 100%, respectively. However, the metabolic simulator gas output is already corrected to STPD using ambient conditions. For this reason, even if perfectly accurate, metabolic devices will measure a lower V̇O_2_ value. For example, if 2 min of data are collected with the simulator running at 82 L·min^−1^ (ATPS) and 2.00 L·min^−1^ in ambient conditions of 740 mmHg, 20.5°C, and 35.6% humidity (STPD = 0.898), the simulator will adjust mass flow to elicit F_E_O_2_ and F_E_CO_2_ of 16.9% and 4.26% so that V̇O_2_ will be 2.00 L·min^−1^ after converting *V̇*_E_ from ATPS to STPD. However, assuming respired air is 100% saturated and nearer body temperature (i.e., 34°C–37°C), the metabolic cart will obtain a STPD factor that is lower than that of the simulator (e.g., 0.819). As a result, the recorded V̇O_2_ will be 1.824 L·min^−1^ or ~10% lower even if the device is perfectly accurate. Interestingly, this error is similar to that of Van Hooren et al. [3], who stated that simulated volumes “were converted from ATPS to STPD” using assumed warm and saturated air. If this was not an error in methodological reporting, this method would lead to a systematic underestimation because it does not consider the effect of differences in “assumed” versus “ambient” conditions. Here, this was accomplished by computing an “STPD ratio” (ambient STPD ÷ assumed STPD) which results in a value greater than 1.0 because the net effect of assuming warm and wet air reduces STPD compared to ambient. Applying a STPD ratio of 1.0964 to the example above gives a V̇O_2_ value measured by the device of 2.00 L·min^−1^ (i.e., 1.824 L·min^−1^ × [0.898 ÷ 0.819]). Notably, the STPD ratio for VO2Master Pro was much lower than that of the COSMED system (see File S1) or than what would have been required for the experiments of Van Hooren et al. [3] because the device was operated in “metabolic simulator mode” (unavailable at the time of Van Hooren et al. [3] publication) which effectively “shuts off” the assumptions and uses instead its internal environmental sensors to derive an STPD factor that is nearer to (but lower than) that of the metabolic simulator. Such differences in sensed environmental variables are expected to produce small errors in comparison between metabolic simulators and the VO2Master system even when operating in “Metabolic Simulator mode”. Here, the error was consistently less than 2%.

Otherwise, with respect to the COSMED Quark system, results are comparable to that of Van Hooren et al. [3]. Their V̇O_2_ measurement percent error ranged from −0.4% to 1.8% among simulated intensities, whereas our mean error was ~3% across intensities. In another study comparing COSMED Quark to simulated V̇O_2_ and V̇CO_2_, Beijst et al. [10] reported an absolute percent difference range of 4%–12%, with absolute percent error (i.e., |%|) increasing with higher simulated intensity. In contrast, the highest absolute percent error we observed was 8% (at 1 L·min^−1^), and the absolute percent error declined as “intensity” increased.

The ATS states that the minimal requirement for accuracy of O_2_ and CO_2_ analyzers is 1%, and, for flow‐measuring devices, it is 3% [5]. Although accuracy values for V̇O_2_, V̇CO_2_, and *V̇*_E_ are not specifically stated, these values indicate that the acceptable range for *V̇*_E_ would be 3%. If FeCO_2_ and VT_E_ were to deviate by 1% and 3%, respectively, then this would translate to an error in the calculated V̇CO_2_ of 3% (V̇CO_2_ = FeCO_2_ × VT_E_ ÷ time). However, because the calculation of V̇O_2_ requires the Haldane transformation (see Equation 1), deviations of FeO_2_ and FeCO_2_ of 1% each (in the same direction) and for VT_E_ of 3% in the opposite direction would yield a maximal acceptable error range of ~9%. Although we and most of the exercise science community are unlikely to agree with 9% as an acceptable level of error, except for the lowest intensity, all measured error values for both devices fell well below these ranges.

### 4.2 Reliability

The measurements of V̇O_2_ and *V̇*_E_ using the VO2Master Pro and COSMED Quark systems showed consistent results across different days, generally less than ±1% variation. A between‐day mean difference of −13, −39, −53, −18 mL·min^−1^ for the COSMED Quark systems and −32, 21, 21, and 61 mL·min^−1^ for the VO2Master Pro devices represent small variations in day‐to‐day measurements during simulated V̇O_2_ of 1.0, 2.0, 3.0, and 4.0 L·min^−1^, respectively. The 95% confidence intervals for COSMED Quark increased from a width of 25 mL·min^−1^ to 91 mL·min^−1^ from simulated V̇O_2_ of 1.0 and 4.0 L·min^−1^, respectively. For the VO2Master Pro devices, the confidence interval width remained stable, equalling 87 mL·min^−1^ at a simulated V̇O_2_ of 1.0 L·min^−1^ and 85 mL·min^−1^ at 4.0 L·min^−1^ of V̇O_2_.

Only a few studies have assessed the between‐day variability of the metabolic measurement systems evaluated herein. In a test–retest design using eight human participants cycling for 5 min at each of 100, 150, 200, and 250 W respectively, Montoye et al. [4] reported mean absolute percent differences in V̇O_2_ measured by the VO2Master of 15.1%, 13.5%, 11.3%, and 6.3%, respectively. For *V̇*_E_, the mean absolute percent error ranged from 5.0% to 13.1%. These values are much greater than our mean absolute percent error values, which did not exceed 3% (when including all repeated trials from all intensities) for either device. In addition, Van Hooren et al. [3] also performed a between‐day comparison of a single VO2Master Pro unit over 4 days and found a mean absolute difference between‐days of 0.22 L·min^−1^ (12.4%) for V̇O_2_ and 6.01 L·min^−1^ (7.7%) for *V̇*_E_, across all intensities. These results are greater than the present study, where the absolute percent error of measured V̇O_2_ varied by 1.2% (or 0.02 L·min^−1^) for V̇O_2_ and 0.3% (or 0.06 L·min^−1^) for *V̇*_E_ across all simulated intensities. At best, we can only speculate as to why our study produced lower test–retest variability. Factors may include differences in days between repeated tests, age of the devices, differences in metabolic simulator manufacturers/technologies and environmental factors. The COSMED Quark system in the present study also exhibited lower between‐day variability, where measured V̇O_2_ varied by 0.10% (or 8 mL·min^−1^) and 0.4% (or 0.003 L·min^−1^) for *V̇*_E_ between‐days. These results confirm that both the VO2Master Pro and COSMED Quark systems provide reproducibility values near or within the American Thoracic Society‐recommended minimum for volume‐ and O_2_‐measuring devices of 3% and 1%, respectively.

Regarding Douglas bag, between‐day measurements of V̇O_2_ displayed absolute percent differences of 11.6%, 1.7%, 1.6%, and 1.9% at 1.0, 2.0, 3.0, and 4.0 L·min^−1^ (see Figure 1), exhibiting a decrease, as well, in absolute error with increased simulated V̇O_2_ outputs across days.

### 4.3 Transferability

Overall, and at each stage, differences in error and percent error in *V̇*_E_ and V̇O_2_ were similar, typically less than a ±1% variation when comparing each of the VO2Master Pro and COSMED Quark analyzers, indicating a high degree of concurrent validity between different (within‐system) devices from the same manufacturer. An inter‐device mean error of 13, 39, 53, and 18 mL·min^−1^ for the COSMED Quark system and 32, −20, ‐21, and −62 mL·min^−1^ for the VO2Master Pro system shows small variation in device‐to‐device measurements of measured V̇O_2_ at 1.0, 2.0, 3.0, and 4.0 L·min^−1^, respectively. Comparable to the between‐day analysis, the COSMED Quark and VO2Master Pro systems displayed a consistent and relatively low error across intensities. Inter‐device variability for V̇O_2_ and *V̇*_E_ has been reported at 13%–14% [4]. However, these comparisons were made using different versions of the device (1.1.1 and 1.2.1) and from nonsimulated conditions. Both these factors are expected to increase variability and could explain why transferability was superior herein.

### 4.4 Human Data

In addition to simulated measurements, a cohort of human participants (*n* = 5) was recruited to determine whether differences between devices with simulated data would be consistent with human experiments. Important to consider is that unlike simulated data, gas exchange and ventilatory responses to exercise at constant‐power output performed at different times (e.g., different days) are subject to additional biological variability that may be due to differences in substrate utilization, circulating hormones, and energy status. With these considerations in mind, the present study discovered no significant differences in measured V̇O_2_ and *V̇*_E_ among all devices at both 100 and 160 W. This is an encouraging outcome for those measuring pulmonary gas exchange with any of these devices or comparing results between analyzers.

### 4.5 Limitations and Other Considerations

Although the simulated V̇O_2_ range of 1–4 L·min^−1^ does largely encompass the aerobic capacity of most individuals in the population, whether results are generalizable to V̇O_2max_ that exceed 4 L·min^−1^ and V̇_Emax_ of 150 L·min^−1^ remains uncertain [11]. In addition, although a brief period of ~30 s was implemented to allow for stabilization of gas exchange and ventilatory variables, the simulated tests were 2 min in length and may not accurately represent the duration of a typical test involving a metabolic cart. To verify the generalizability of our findings to longer tests, all devices underwent an additional round of simulated experiments performed at the median of the four simulated intensities (i.e., V̇O_2_ of 2.5 L·min^−1^, *V̇*_E_ of 75 L·min^−1^ and *B*_F_ of 30 breaths per minute) and were assessed and for a period of 20 min (1200 s). When assessing the COSMED Quark system across three, 90 s time periods (i.e., 30–120 s, 600–690 s, and 1110–1200 s) both devices exhibited stable measurements across the entirety of the test as evidenced by a minuscule change in V̇O_2_ from the 2nd to 20th minutes of 1 mL·min^−1^·min^−1^ and ‐1 mL·min^−1^·min^−1^ for COSMED Quark #1 and 2, respectively. In contrast, both VO2Master Pro devices demonstrated positive rises netting 110 and 170 mL·min^−1^, respectively. Notably, the rises were not perfectly linear and resembled more of an exponential response, with a rise that diminished between the latter two timepoints. This behavior was confirmed in 5 out of 6 repeated trials and appeared to be due to a fall in FeO_2_ that averaged ~0.013 units. Variably, the “drift” either brought the V̇O_2_ closer to or further from the simulated value depending on the V̇O_2_ registered in the 90 s preceding Minute 2. That said, in “steady‐state”, the percent error averaged 1.6% (range: −3.1%–3.4%) and was more consistent with the 2 min simulated trials. Finally, the human experiments elicited submaximal V̇O_2_ and utilized a small sample size. Whether differences would have emerged at higher intensities and with more individuals is unlikely but possible.

All experiments were performed in a laboratory setting. As a portable device, the VO2Master Pro system may also be used outside of the laboratory where environmental factors (e.g., wind, changes in temperature, humidity, altitude, etc.) could impact validity, reliability, and inter‐device error. Thus, results of this study should not be assumed to hold true outside of the laboratory and in “real‐world” conditions.

### 4.6 Practical Observations

We noted that VO2Master Pro may exhibit noisy and nonsensical values of F_E_O_2_ if it was calibrated by connecting the device directly to the syringe without using the recommended clear hose adaptor. Specifically, fluctuations in F_E_O_2_ were observed to intermittently spike, resulting in sporadic and inaccurate values during preliminary trials. Subsequent inquiry with the manufacturer elucidated that such occurrences had not been previously observed, and it was suggested that the abrupt airflow experienced during the end of exhalation from the calibration syringe may potentially disrupt or “shock” the oxygen sensor, causing erratic readings.

## 5 Conclusions

Using the VacuMed metabolic simulator to simulate human pulmonary gas exchange and ventilation, we compared the expected values of *V̇*_E_, *B*_F_, *V*_T_, V̇O_2_, and V̇CO_2_ variables at four exercise “intensities” (1.0, 2.0, 3.0, and 4.0 L·min^−1^) with the measured values of two metabolic measurement systems (COSMED Quark CPET and VO2Master Pro). This study demonstrated that the error of *V̇*_E_ and V̇O_2_ during simulated breathing is generally < 3% and is consistent between‐days and between‐devices within a simulated V̇O_2_ range of 1–4 L·min^−1^. Notably, the overall relative error of measured V̇O_2_ for the VO2Master System averaged ±0.6% (SD of 3.0%), which is similar to reported error rates (± 1%) for the VacuMed simulator. Findings also indicate low variability concerning between‐day accuracy for all four devices. Together, when in good operating condition and meticulously operated, both systems appear valid, reliable, and transferable and are, therefore, suitable for within‐day, between‐day, and between‐device use in a laboratory setting.

## Author Contributions

J.S.T., N.A.G., R.F., and D.A.K. designed the study protocol. J.S.T., N.A.G., and R.F. contributed to the data collection. J.S.T. and R.F. treated and analyzed the results. J.S.T., N.A.G., R.F., and D.A.K. composed the journal article.

## Ethics Statement

The protocol was reviewed and approved by the Institutional Research Ethics Review Board for human participants (WREM: 123924) and complied with the Declaration of Helsinki.

## Consent

All participants were conscious of their right to withdraw at any time from the study and gave written informed consent.

## Conflicts of Interest

The authors declare no conflicts of interest. The results of the study are presented clearly, honestly, and without fabrication, falsification, or inappropriate data manipulation.

## Supporting information


Appendix S1.

## Data Availability

All recorded and computed data from this study are available in Appendix S1.
